# Reinstatement of "germinal epithelium" of the ovary

**DOI:** 10.1186/1477-7827-4-42

**Published:** 2006-08-21

**Authors:** Takashi Nishida, Naoyo Nishida

**Affiliations:** 1 Oita-Ken Saiseikai Hita Hospital, Social Welfare Organization, Saiseikai Imperial Gift Foundation Inc., 643-7 Miwa, Hita-shi, 877-1292, Japan; 2 Department of Pathology, Kurume University School of Medicine, 67 Asahi-machi, Kurume-shi, 830-0011, Japan

## Abstract

**Background:**

The existing dogma that the former term ovarian "germinal epithelium" resulted from a mistaken belief that it could give rise to new germ cells is now strongly challenged.

**Discussion:**

Two years ago, a research group of the University of Tennessee led by Antonin Bukovsky successfully demonstrated the oogenic process from the human ovarian covering epithelium now commonly called the ovarian surface epithelium. They showed the new oocyte with zona pellucida and granulosa cells, both originated from the surface epithelium arising from mesenchymal cells in the tunica albuginea, and stressed that the human ovary could form primary follicles throughout the reproductive period. This gives a big impact not only to the field of reproductive medicine, but also to the oncologic area. The surface epithelium is regarded as the major source of ovarian cancers, and most of the neoplasms exhibit the histology resembling müllerian epithelia. Since the differentiating capability of the surface epithelium has now expanded, the histologic range of the neoplasms in this category may extend to include both germ cell tumors and sex cord-stromal cell tumors.

**Summary:**

Since the oogenic capability of ovarian surface cells has been proven, it is now believed that the oocytes can originate from them. The term "germinal epithelium", hence, might reasonably be reinstated.

## Background

Unlike the avian ovary, where the follicles containing a large yolky egg look like a cluster of yellowish grapes, the human ovary is solid in structure, amygdaloid in shape, grayish white in color, and the follicles are usually invisible through the surface. Since the intraovarian follicle was firstly identified by Reinier De Graaf in 1672 [1], the origin of human oocyte has been disputed. Two opposing views have been known regarding the source of oocytes: (1) that they arise in the yolk sac [2-5], or (2) that they arise in the gonadal tissue itself [6-11]. The former view had gotten public consensus until the astonishing works by Bukovsky et al. on the capability of ovarian covering tissue to produce new oocytes in adult human females were published [12-15].

A study of the histogenesis of ovarian components is essential to understand the oncogenesis of ovarian neoplasms. Since the ovarian covering tissue has now been revealed to have oogenic capability, the surface epithelium might be accountable as a source of germ cell tumors and sex cord-stromal cell tumors, as well as neoplasms exhibiting the müllerian histology.

## Discussion

The nature of covering epithelium of the ovary is intriguing. Although only a part of peritoneal mesothelium, it proliferates to repair the minor trauma due to the ovulation, and thus has occasionally tumorigenic potential. The concept that the epithelium simulates the müllerian form in tumor formation has developed by degrees and now becomes a firm policy in the classification of ovarian cancer [16]. Although mucinous tumors are also placed in the common epithelial category, it is still disputed whether the origin of a certain group of mucinous neoplasms composed of intestinal type cells arose from germ cells, namely monophyletic teratomata.

In our previous experimental study, in which a chemical carcinogen, 7,12-dimethylbenz [a]anthracene (DMBA), was directly applied to the rat ovarian surface, an ovarian cancer was observed in about half of the DMBA-treated rats. The histology of the induced tumors also simulated the epithelia of rat genital tracts. In the experiment, however, an unexplainable cancer composed of heterologous osteoid tissue was observed [17]. At that time, if the covering tissue of the ovary were not called "surface epithelium", but named "germinal epithelium" instead, the tumor might have been classified as a teratomatous osteosarcoma.

## Summary

From the capability of ovarian covering tissue to produce oocytes, the term "germinal epithelium" might be reinstated.
